# Supplementary material for: Language and beliefs in relation to noma: a qualitative study, northwest Nigeria
Source: PLoS Negl Trop Dis. 2020 Jan 23;14(1):e0007972. doi: 10.1371/journal.pntd.0007972 (PMC6999908; doi:10.1371/journal.pntd.0007972)
Supplement: S1 Text — (DOCX) [file pntd.0007972.s001.docx]

**S1 Focus group discussion guide**

**Introduction**

•Thank the participants for agreeing to take part in this research, introduce yourself.

•Create a relaxed atmosphere; offer the participants something to drink when this is possible.

•Tell the group *“I (We) would like to talk to you about noma, your beliefs about this disease and the words you use to describe it. This discussion will contribute to a better understanding of how people living with noma experience the disease. The interview will take approximately 45 - 60 minutes with anyone in the group deciding to leave at any time if they no longer want to contribute at any time without any consequences.”*

•Make sure the group participants have been informed about the study and have consented to participate in the research. NOTE: Turn on the recorder and test it is recording.

| **Topic** | **Specific Questions** | **Prompts and Notes** |
| --- | --- | --- |
| Introduction | - Study aim  - Why invited to participate  - Consent and respect within the group | Answer any questions group has about study |
| Language used to describe noma | - Show picture of noma patient and ask group- if this person was walking in your village, what name would you use to describe the disease they had?  -Ask what their community would call the disease?  - What the names mean?  - Where the names come from? | Specific names, ask about origins of names, ask each group member what name it is called in their village, try and see if there are variations. |
| Perception of noma | - Ask caretakers what other people say about this disease?  - What do community members know about disease?  - Is it a common disease? | Ask about community and personal perceptions of the disease. |
| Beliefs | - Where does the disease come from?  - What causes the disease?  - Risk factors for disease?  - How best to treat disease? | Omen, spell, come from the wind, witchcraft. |
