# Supplementary material for: Language and beliefs in relation to noma: a qualitative study, northwest Nigeria
Source: PLoS Negl Trop Dis. 2020 Jan 23;14(1):e0007972. doi: 10.1371/journal.pntd.0007972 (PMC6999908; doi:10.1371/journal.pntd.0007972)
Supplement: S2 Text — (DOCX) [file pntd.0007972.s002.docx]

**S2 In-depth interview guide**

The key informant interviews will be conducted in a setting that minimises the influence of the researcher and encourages the interviewee to discuss noma, and their role in the project.

**Introduction**

•Thank the participant for agreeing to take part in this research.

•Introductions- explain who you are and share some of your experience.

•Create a relaxed atmosphere; offer the participant something to drink when this is possible.

•Tell each participant: *“I (we) would like to talk to you about your experiences of noma and your role at the hospital. We would like to hear your stories, what you think about the disease, what works well for treatment and the challenges you face. This interview will contribute to a better understanding of noma and the work occurring at the Noma Children’s Hospital in Sokoto. The interview will take approximately 30 minutes and can be stopped by you at any time without any consequences. If you would like to continue there is a form we have to complete to check that you have all the information you need before we start. Would you like to continue? Would you be happy for us to record this conversation for our records?*

•Make sure the participant has been informed and has consented to participate in the research study. NOTE: Turn on the recorder and test it is recording.

Questions should be adapted where necessary, but can follow the below themes:

1. Discuss patient flow thorough the noma program.
2. What noma stage patients are at when they are included in to the program?
3. How long on average after onset do patients come to hospital?
4. What would the best terms be to use in the questionnaire to describe noma?
5. What beliefs staff have about noma?
6. What beliefs about noma have staff heard from patients?
